# Surface‐Amorphous and Oxygen‐Deficient Li_3_VO_4−*δ*_ as a Promising Anode Material for Lithium‐Ion Batteries

**DOI:** 10.1002/advs.201500090

**Published:** 2015-06-10

**Authors:** Liang Chen, Xiaolei Jiang, Nana Wang, Jie Yue, Yitai Qian, Jian Yang

**Affiliations:** ^1^ School of Chemistry and Chemical EngineeringShandong UniversityJinan250100P. R. China; ^2^ Hefei National Laboratory for Physical Science at MicroscaleDepartment of ChemistryUniversity of Science and Technology of ChinaHefei230026P. R. China

**Keywords:** Li_3_VO_4_, lithium‐ion batteries, oxygen deficient

## Abstract

**Surface‐amorphous and oxygen‐deficient Li_3_VO_4−*δ*_** **synthesized by simple annealing of Li_3_VO_4_ powders in a vacuum** shows great enhancements in both reversible capacity and coulombic efficiency for the first discharge/charge without delicate size control and carbon coating. The results are associated with the improved charge‐transfer kinetics caused by the amorphous surface of Li_3_VO_4−*δ*_.

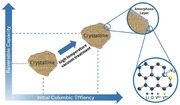

Lithium‐ion batteries (LIBs) as a reliable and high‐efficient energy‐storage device, have achieved great successes in a variety of applications, such as portable electronics, electric vehicles, stationary grid storage, and so on. As the developing of these applications, conventional anode material, graphite, becomes more and more difficult to satisfy their ever‐growing needs on energy density, in view of its low theoretical capacity.^1^, ^2^ Thus, the alloy‐type anodes, like silicon,^3^, ^4^ tin,^5^, ^6^ etc., and the conversion‐type anodes,^7^ usually transitional metal oxides,^8^ are being explored as potential candidates for anode materials, due to their high capacities. But they have to experience a severe volume change during the lithiation/delithiation process, which often leads to particle pulverization and capacity degradation. This issue could be greatly mitigated in intercalation‐type anode materials, bringing good cycling stability and high rate capability. Unfortunately, the choices of these anode materials are quite limited and most of them have their own shortages. Graphite, as stated above, is limited by its low theoretical capacity and safety issues. Li_4_Ti_5_O_12_, another important member of this family, has the insertion of Li^+^ occur at ≈1.55 V (vs Li/Li^+^), thus halving the overall cell voltage. Moreover, its small theoretical capacity (≈175 mA h g^−1^) further reduces its merit.^9^, ^10^

Different from vanadium oxides extensively reported as cathode materials,^11^, ^12^, ^13^, ^14^ Li_3_VO_4_ as a new‐emerging anode material based on intercalation reactions, has a moderate working potential lower than Li_4_Ti_5_O_12_, but higher than graphite.^15^, ^16^ This offers the Li_3_VO_4_‐based anode a better safety than carbon and a larger output voltage in a full cell than Li_4_Ti_5_O_12_. Moreover, its theoretical capacity (≈394 mA h g^−1^)^16^, ^17^ is also higher than those of carbon and Li_4_Ti_5_O_12_. Its structure analogous to Li_3_PO_4_, a solid ionic conductor, further adds its value as an anode material.^18^ In spite of these advantages, Li_3_VO_4_ has to face the challenges from poor electronic conductivity and low coulombic efficiency during the first charge/discharge. The issues could be addressed by size control into nanometers and surface coating by carbon, both of which have been demonstrated in a large number of works including in Li_3_VO_4_.^19^, ^20^, ^21^ However, the usage of nanostructured materials would induce additional negative effects, such as low tap density, large irreversible capacity loss, and large interparticle resistance.^22^ Surface coating with carbon would greatly reduce the volumetric energy density and affect the processing performances of anode materials for electrode in practical applications.^23^, ^24^

Here, another totally different strategy has been developed to improve the electrochemical properties of Li_3_VO_4_, using irregular powders without particular size and shape controls as a model. The obtained surface‐amorphous and oxygen‐deficient Li_3_VO_4_ (Li_3_VO_4−*δ*_), synthesized by a simple annealing of Li_3_VO_4_ powders in vacuum, shows great improvements in reversible capacity and coulombic efficiency for the first discharge/charge process simultaneously. These improvements could be ascribed to enhanced charge‐transfer kinetics of Li_3_VO_4−*δ*_, where its unique amorphous surface rich in structure defects is vital in comparison to Li_3_VO_4_. The charming aspects of this method lie in that it realizes the electrochemical improvements of electrode materials via a new strategy totally different from nanostructure engineering and carbon coating. Moreover, this method could effectively avoid their negative consequences. Most important, this synthesis is convenient and cost‐effective, then particularly suitable for the mass production of high‐performance electrode materials.

Li_3_VO_4−*δ*_ was prepared by annealing of Li_3_VO_4_ powders at 500 °C in vacuum for 1 h. **Figure** **1**A,B shows the SEM images of Li_3_VO_4_ and Li_3_VO_4−*δ*_. Both of them are composed of irregular particles with a broad size distribution, which implies that the annealing in vacuum does not change the morphology and size of these particles. The close‐up check on the particles, particularly at the edges, brings up the difference between them. As shown in Figure 1C,D, an amorphous layer of ≈5 nm is present on the particle surface of Li_3_VO_4−*δ*_, but absent on that of Li_3_VO_4_. In order to clarify this difference on the particle surface, XPS spectra were measured for both of them. Although the survey spectra of Li_3_VO_4_ and Li_3_VO_4−*δ*_ (Figure S1, Supporting Information) are almost identical, their high‐resolution spectra of V 2p are very different. The spectra of V 2p in Li_3_VO_4_ (Figure 1E) could be well fitted by a doublet of V 2p_1/2_ and V 2p_3/2_ from V^5+^, along with an X‐ray satellite of O 1s.^25^ That of V 2p in Li_3_VO_4−*δ*_ (Figure 1F) has to be deconvoluted into two doublets of V 2p_1/2_ and V 2p_3/2_ from V^4+^ and V^5+^,^25^ as well as an X‐ray satellite of O 1s. This result indicates that the annealing in vacuum induces the reduction of V^5+^ to V^4+^. Along with this reduction, oxygen would be generated and released from the particles, resulting in the appearance of oxygen vacancies and the formation of Li_3_VO_4−*δ*_. Taking the results from HRTEM images into account, it is likely that the conversion from V^5+^ to V^4+^ results in huge lattice stress, structure rearrangement, and gradual amorphization. Because the particle surface is highly activated, easy for oxygen to escape from the interior, and well exposed to a high temperature, the amorphous layer would be preferentially formed on the surface. The correlation of the amorphous layer and V^4+^ distribution in Li_3_VO_4−*δ*_ is validated by the depth analysis based on XPS spectra. It is conducted by exposing Li_3_VO_4−*δ*_ to Ar^+^ sputtering. As indicated in Figure S2 (Supporting Information), the content of V^4+^ decreases with the sputtering time, which becomes even more apparent in terms of V^4+^/V^5+^. The results indicate that V^4+^ in Li_3_VO_4−*δ*_ concentrates on the surface, likely in the amorphous layer. Because the amorphous structure is less rigid than its crystalline counterpart, it would show a lower energy barrier and a better tolerance to the interface stress induced by lithium insertion/extraction.^26^, ^27^

**Figure 1:**
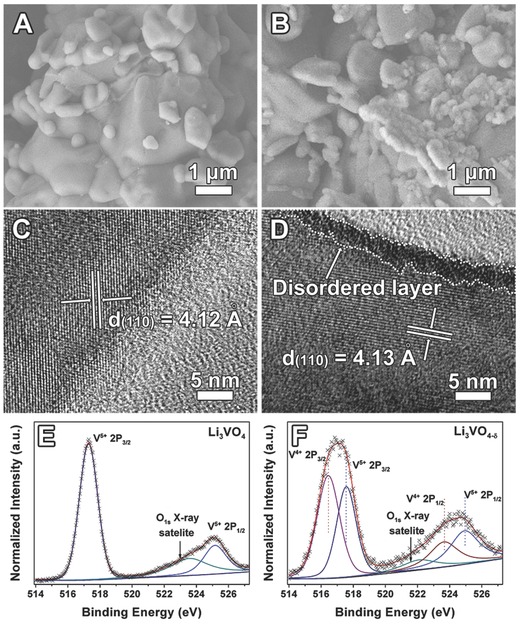
SEM and HRTEM images of A,C) Li_3_VO_4_ and B,D) Li_3_VO_4−*δ*_. XPS spectra of V 2p for E) Li_3_VO_4_ and F) Li_3_VO_4−*δ*_.

Compared with the significant increase of V^4+^ on the amorphous surface, it is much less in the crystalline core of Li_3_VO_4−*δ*_ powders. As shown in **Figure** **2**A, the strong and narrow diffraction peaks indicate the high crystallinity of Li_3_VO_4_ and Li_3_VO_4−*δ*_. All the diffraction peaks in both patterns could be indexed to orthorhombic‐phase Li_3_VO_4_ (JCPDS No. 38‐1247) without any impurities, indicating that the annealing in vacuum does not alter the crystal structure of Li_3_VO_4_. But there is a tiny lattice expansion from Li_3_VO_4_ to Li_3_VO_4−*δ*_, which is supported by the slight shift of the diffraction peaks to the low angles (the inset of Figure 2A). This shift has been observed for multiple times in different batches of the powders before and after the annealing, excluding the measurement errors. This lattice expansion might be related to the increase of V^4+^ in the crystalline core of Li_3_VO_4−*δ*_, because V^4+^ has a larger ionic radii than V^5+^ (V^4+^: 58 pm; V^5+^: 54 pm).^28^ Accompanied with the increase of V^4+^ ions, the number of oxygen vacancies associated with V^4+^, also goes up. Since Li_3_VO_4_ consists of corner‐sharing LiO_4_ and VO_4_ tetrahedrons (Figure 2B), all the oxygen atoms are supposed to be there for the connection of LiO_4_ and VO_4_ tetrahedrons. So, the formation of oxygen vacancies at these sites would also offer more spaces for Li^+^ to diffuse and benefit the improvement of electrochemical properties.

**Figure 2:**
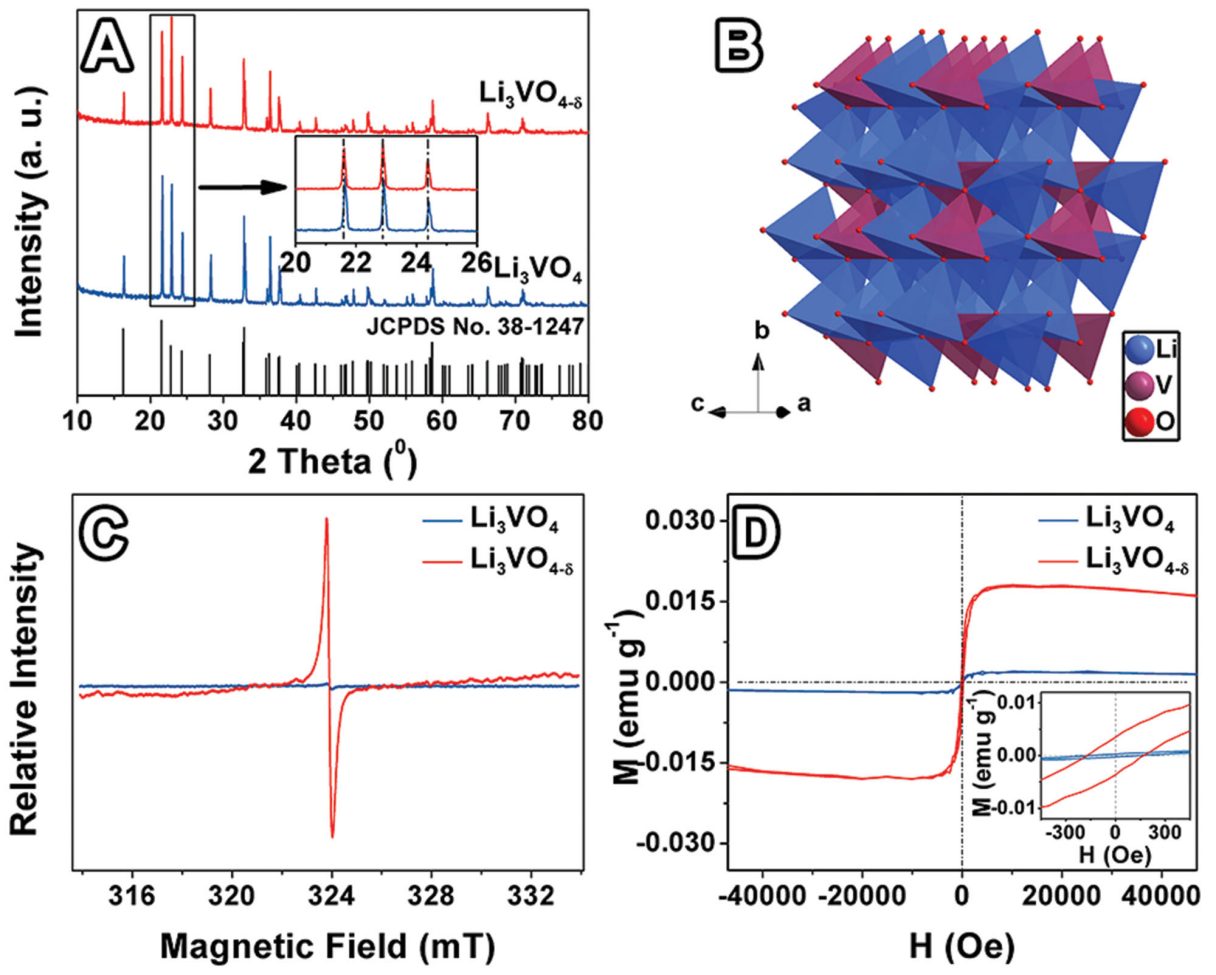
A) XRD patterns of Li_3_VO_4_ and Li_3_VO_4−*δ*_. B) Crystal structure of orthorhombic‐phase Li_3_VO_4−*δ*_. C) EPR spectra and D) magnetic measurements of Li_3_VO_4_ and Li_3_VO_4−*δ*_.

All these V^4+^ species in Li_3_V_4−*δ*_, no matter whether they are in the amorphous surface or in the crystalline core, would lead to a large number of unpaired electrons that could be easily detected by electron paramagnetic resonance (EPR) spectra and magnetic measurements. As shown in Figure 2C, there is a great increase in the EPR signal from Li_3_VO_4_ to Li_3_VO_4−*δ*_, corresponding to the unpaired 3d^1^ electrons of increased V^4+^ species in Li_3_VO_4−*δ*_. Due to the high concentration and strong spin‐coupling of these defects, the hyperfine structure of V^4+^ is absent in the spectra.^29^ The similar result is also obtained from magnetic properties. As presented in Figure 2D, the saturation magnetization (*M*_s_ ≈ 0.017 emu g^−1^) and remanent magnetization (*M*_r_ ≈ 0.003 emu g^−1^) of Li_3_VO_4−*δ*_ are much higher than those of Li_3_VO_4_.

The electrochemical performances of Li_3_VO_4_ and Li_3_VO_4−*δ*_ are evaluated in a half‐cell configuration. All the cells are cycled between 0.2 and 3.0 V at a given current density. Although Li_3_VO_4_ and Li_3_VO_4−*δ*_ exhibit similar electrochemical behaviors (Figure S3, Supporting Information), Li_3_VO_4−*δ*_ presents the lithium‐storage performances much better than Li_3_VO_4_. As shown in **Figure** **3**A, the first discharge/charge capacity of Li_3_VO_4_ at 200 mA g^−1^ is 280/162 mA h g^−1^, corresponding to a coulombic efficiency of 58%. After the degradation during the first several cycles, its capacity would stabilize at ≈110 mA h g^−1^ after 200 cycles (Figure 3B and Figure S4, Supporting Information). Compared to the case of Li_3_VO_4_, there are significant enhancements for Li_3_VO_4−*δ*_ in terms of the coulombic efficiency at the first cycle and the reversible capacity after 200 cycles. Specifically, the discharge/charge capacity of Li_3_VO_4−*δ*_ at the first cycle increases to 416/326 mA h g^−1^, giving a coulombic efficiency of 78%. After 200 cycles at 200 mA g^−1^, the specific capacity of Li_3_VO_4−*δ*_ is promoted to 286 mA h g^−1^ (Figure S4, Supporting Information), nearly three times better than those of Li_3_VO_4_. These results clearly demonstrate the advantages of Li_3_VO_4−*δ*_ in both the capacities and the coulombic efficiency.

**Figure 3:**
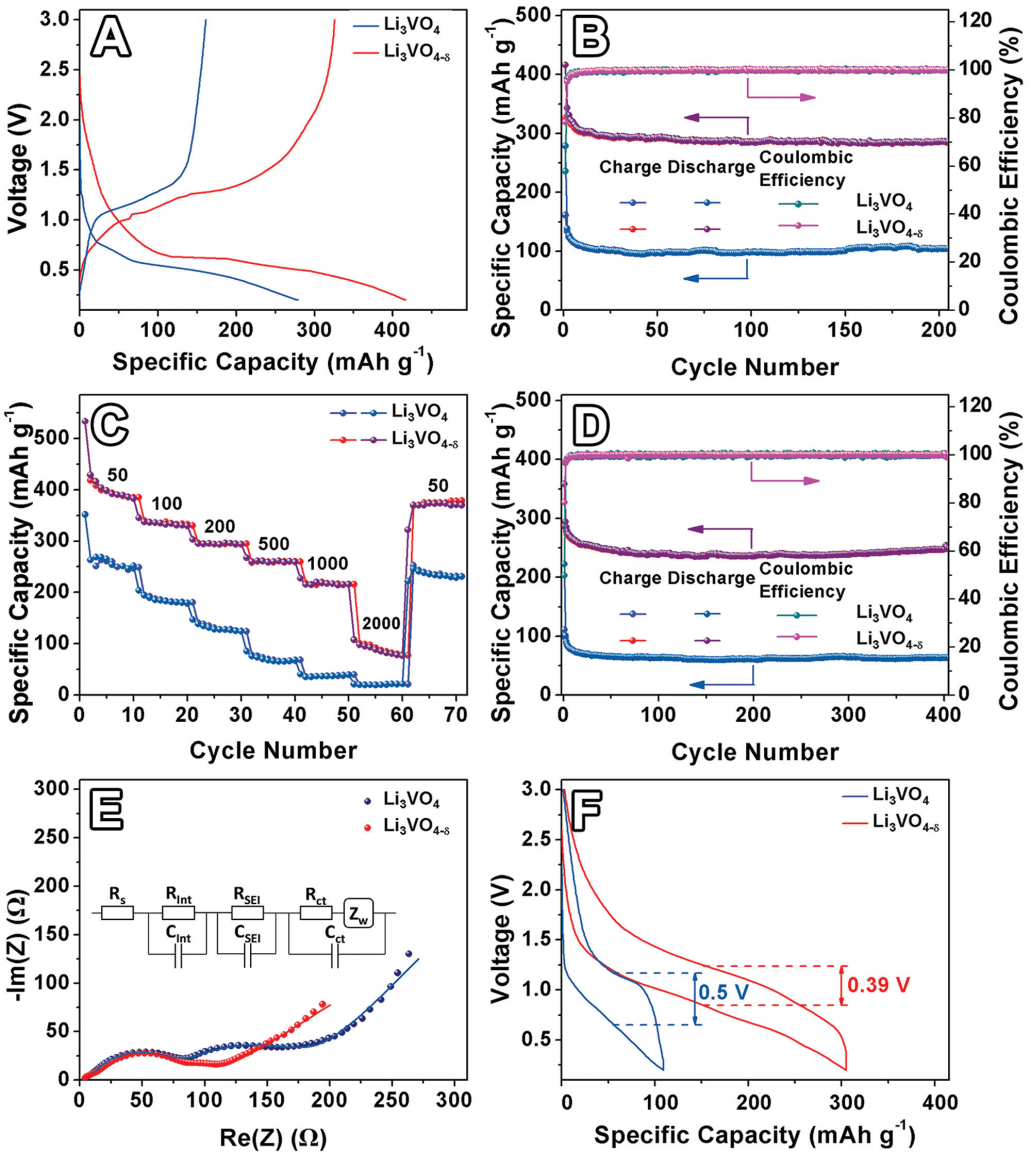
A) The first galvanostatic charge–discharge profiles and B) cycling performances and coulombic efficiencies of Li_3_VO_4_ and Li_3_VO_4−*δ*_ at a current density of 200 mA g^−1^. C) Rate performances of Li_3_VO_4_ and Li_3_VO_4−*δ*_. D) Cycling performances and coulombic efficiencies of Li_3_VO_4_ and Li_3_VO_4−*δ*_ at a current density of 500 mA g^−1^. E) Nyquist plots of Li_3_VO_4_ and Li_3_VO_4−*δ*_ at 0.75 V after five cycles. F) Galvanostatic charge–discharge profiles of Li_3_VO_4_ and Li_3_VO_4−*δ*_ at tenth cycle at a current density of 200 mA g^−1^.

The same conclusion could also be concluded from the rate performance. As illustrated in Figure 3C, the reversible capacity of Li_3_VO_4−*δ*_ is 380 mA h g^−1^ at 50 mA g^−1^, 335 mA h g^−1^ at 100 mA g^−1^, 300 mA h g^−1^ at 200 mA g^−1^, 260 mA h g^−1^ at 500 mA g^−1^, 215 mA h g^−1^ at 1000 mA g^−1^, or 90 mA h g^−1^ at 2000 mA g^−1^, all of which are much higher than their counterparts from Li_3_VO_4_. As the current density comes back to 50 mA g^−1^, the specific capacity of Li_3_VO_4−*δ*_ returns to 375 mA h g^−1^, indicating a good electrochemical reversibility. These data are also very close to its theoretical capacity (394 mA h g^−1^). The superior rate capability of Li_3_VO_4−*δ*_ to Li_3_VO_4_ could be illustrated in another way. As shown in Figure S5 (Supporting Information), it takes ≈31 min for Li_3_VO_4−*δ*_ to be charged to 260 mA h g^−1^, but at least 5 h for Li_3_VO_4_ to reach the same capacity. The faster charging rate of Li_3_VO_4−*δ*_, ≈10 times than that of Li_3_VO_4_, confirms again the better rate capability of Li_3_VO_4−*δ*_. Even at a high rate, Li_3_VO_4−*δ*_ still keeps an excellent cycling stability. As described in Figure 3D and Figure S4 (Supporting Information), Li_3_VO_4−*δ*_ presents a reversible capacity of 247 mA h g^−1^ after 400 cycles at a current density of 500 mA g^−1^, much higher than Li_3_VO_4_ (≈64 mA h g^−1^). It should be noted that these data are obtained without particular control in particle size and carbon coating for Li_3_VO_4−*δ*_. Because these controls could improve the capacity and reversibility, the further improvement for Li_3_VO_4−*δ*_ by the combination of all these tactics are on the way.

EIS spectra of Li_3_VO_4_ and Li_3_VO_4−*δ*_ are measured at 0.75 V after five cycles to gain the insights about the excellent performance of Li_3_VO_4−*δ*_. As shown in Figure 3E, both the spectra consist of several depressed semicircle in the region of high‐to‐medium frequencies followed with a slope with an angle of 45° in the region of low frequencies. The equivalent circuit shows that the SEI‐related resistance (*R*_SEI_) and the charge‐transfer resistance (*R*_ct_) of Li_3_VO_4−*δ*_ decrease to 10.8 and 66.9 Ω from 68.4 and 113 Ω of Li_3_VO_4_. Both of them could be associated with the amorphous surface in Li_3_VO_4−*δ*_, because its isotropic and disorder nature could relax the high strain caused by Li‐ion insertion, and facilitate the Li‐ion diffusion via percolation pathways.^30^, ^31^ The latter is also reflected by the Li‐ion diffusion coefficient increase from 2.47 × 10^−12^ cm^2^ s^−1^ of Li_3_VO_4_ to 3.82 × 10^−12^ cm^2^ s^−1^ of Li_3_VO_4−*δ*_ (Figure S6, Supporting Information). All the results would improve the reaction kinetics and achieve the performance enhancements of Li_3_VO_4−*δ*_. The similar results have already been reported for various TiO_2−*x*_ nanocrystals as an anode material.^32^, ^33^, ^34^ Chen and co‐workers attributed this phenomenon to the “built‐in electric field” across the interface between the amorphous layer and the crystalline core.^33^ Wang and Chen thought that this enhancement came from the disordered surface and Ti^3+^ species via pseudocapacitive lithium storage.^34^ Most important, the voltage gap between the redox couple in Li_3_VO_4−*δ*_ (≈0.39 V) is smaller than that of Li_3_VO_4_ (≈0.5 V), as illustrated in Figure 3F. The small voltage gap implies a decreased transport resistance and a reduced polarization in lithium extraction/insertion, facilitating the improvement of electrochemical performances.

In summary, oxygen‐deficient Li_3_VO_4_ synthesized by a simple annealing of Li_3_VO_4_ in vacuum, is made of a crystalline core and an amorphous surface rich in V^4+^ ions/oxygen vacancies. Compared with the case of Li_3_VO_4_, the presence of this amorphous surface greatly enhances the electrochemical performances of Li_3_VO_4−*δ*_, in both reversible capacity and coulombic efficiency for the first discharge/charge. The results could be correlated to the improved charge‐transfer kinetics of Li_3_VO_4−*δ*_, due to its amorphous surface. This simple, convenient, and cost‐effective method allows it to be promising for the mass production of high‐performance anode materials.

## Supporting information

As a service to our authors and readers, this journal provides supporting information supplied by the authors. Such materials are peer reviewed and may be re‐organized for online delivery, but are not copy‐edited or typeset. Technical support issues arising from supporting information (other than missing files) should be addressed to the authors.

Supplementary
