# Supplementary material for: Surface‐Amorphous and Oxygen‐Deficient Li3VO4−δ as a Promising Anode Material for Lithium‐Ion Batteries
Source: Adv Sci (Weinh). 2015 Jun 10;2(9):1500090. doi: 10.1002/advs.201500090 (PMC5033021; doi:10.1002/advs.201500090)
Supplement: Supplementary file 1 — Supplementary [file ADVS-2-0a-s001.pdf]

Copyright WILEY-VCH Verlag GmbH & Co. KGaA, 69469 Weinheim, Germany, 2015.

ADVANCED SCIENCE
Open Access

Supporting Information

for *Adv. Sci.*, DOI: 10.1002/advs. 201500090

Surface-Amorphous and Oxygen-Deficient $Li_3VO_{4-\delta}$ as a Promising Anode Material for Lithium-Ion Batteries

*Liang Chen, Xiaolei Jiang, Nana Wang, Jie Yue, Yitai Qian, and Jian Yang**

Supporting Information

Surface-Amorphous and Oxygen-Deficient $Li_3VO_{4-\delta}$ as a Promising Anode Material for Lithium Ion Batteries

*Liang Chen, Xiaolei Jiang, Nana Wang, Jie Yue, Yitai Qian and Jian Yang**

Experimental Section

Synthesis of Li_3VO_4 and $Li_3VO_{4-\delta}$: Li_3VO_4 was prepared by a simple solid-state reaction between NH_4VO_3 and Li_2CO_3. Briefly, 0.412 g NH_4VO_3 and 0.395 g Li_2CO_3 powders were mixed thoroughly by ball-milling, then pressed into a tablet. The tablet was calcined at 550 ^oC for 3 h and followed by another calcination at 700 ^oC for 3 h in air at a heating rate of 5 ^oC min^{-1}. The as-obtained Li_3VO_4 was grounded into powders and annealed at 500 ^oC in vacuum for 1 h, generating $Li_3VO_{4-\delta}$ powders.

Sample Characterization: X-ray powder diffraction (XRD) patterns were achieved on a Bruker D8 advanced X-ray diffractometer with Cu Kα radiation (λ = 1.5418 Å). SEM and TEM images were acquired from a field-emission scanning electron micro- scope (SEM, SUPRATM 55) or a transmission electron microscope (TEM, JEM-1011) working. X-ray photoelectron spectra (XPS) were measured on an X-ray photoelectron spectrometer (ESCALAB 250) by referencing the C 1s peak to 284.6 eV. The electron paramagnetic resonance (EPR) spectra were acquired on a JEOL JES FA200 EPR spectrometer at room temperature. The magnetization was characterized by a superconducting quantum interference device (SQUID, quantum design MPMS XL-7) magnetometer at 77K.

Electrochemical Measurements: The working electrode was composed of 70 wt% active material, 20 wt% acetylene black as a conductive additive, and 10 wt% sodium salt of caboxymethyl cellulose (CMC) as a binder. The powders were dispersed in several droplets of deionized water and milled for 30 minutes, giving a black slurry. The slurry was bladed on a copper foil and dried in vacuum at 60 ^oC. The resultant films were pressed and punched into the discs with a diameter of 12 mm. The typical loading density of the active material on the disc was ~2 mg cm^{-2}. Then, the disc was assembled with a lithium foil as the counter and reference electrode, a mixture of 1 M LiPF6 in ethylene carbonate (EC)-ethyl methyl carbonate (EMC)-dimethyl carbonate (DMC) (1:1:1 by volume) as the electrolyte, and a Celgard 2300 microporous membrane as the separator in an argon-filled glove box (Mikrouna, Super 1220/750/900) for CR2032 coin cells. Galvanostatic discharge-charge cycles of cells were performed at room temperature by Land CT2001A battery cycler (Xinnuo, Wuhan

China) in a voltage range of 3.0-0.2 V at different current densities. Cyclic voltammetry (CV) was evaluated by a LK2005A electrochemical workstation over 0.2-3.0 V at various scan rates. Electrochemical impedance spectra (EIS) were acquired from an Autolab PGSTAT302N electrochemical workstation.

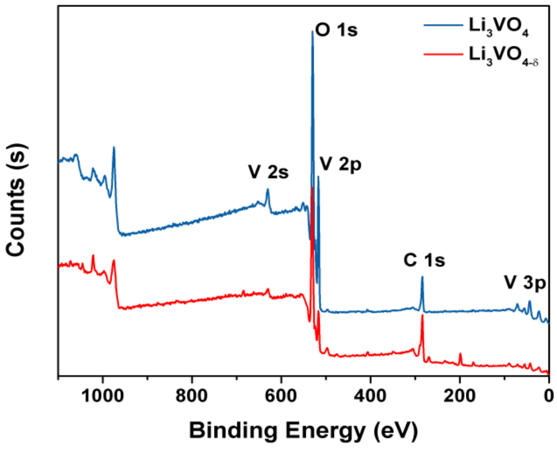

Figure S1. XPS survey spectra pristine Li_3VO_4 and $Li_3VO_{4-\delta}$

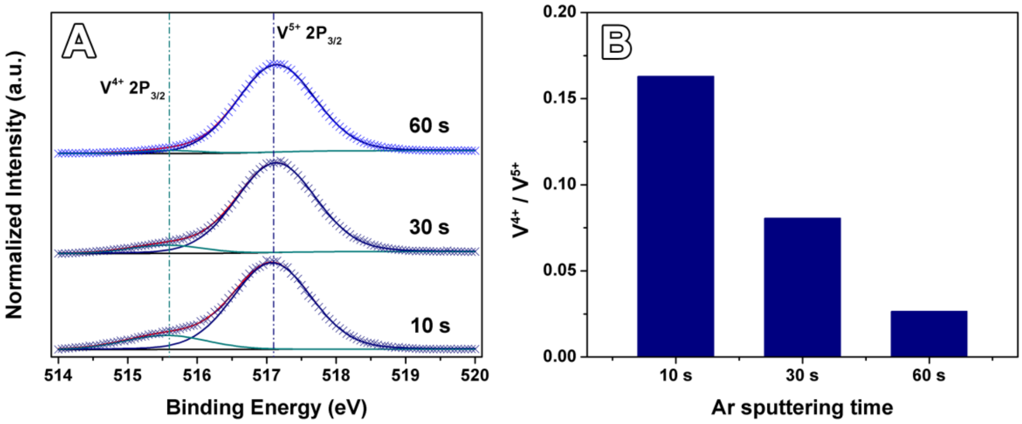

Figure S2. The evolution of V 2p spectra and V^{4+}/V^{5+} with Ar^+ sputtering time.

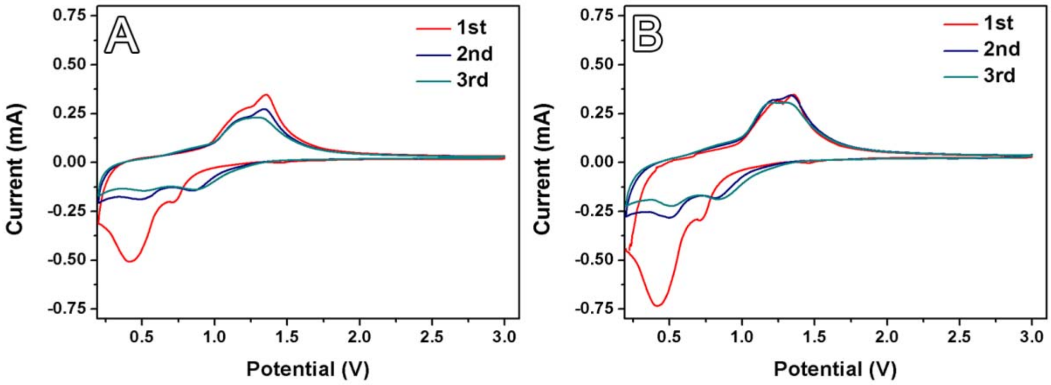

Figure S3. CV curves of Li_3VO_4 and $Li_3VO_{4-\delta}$.

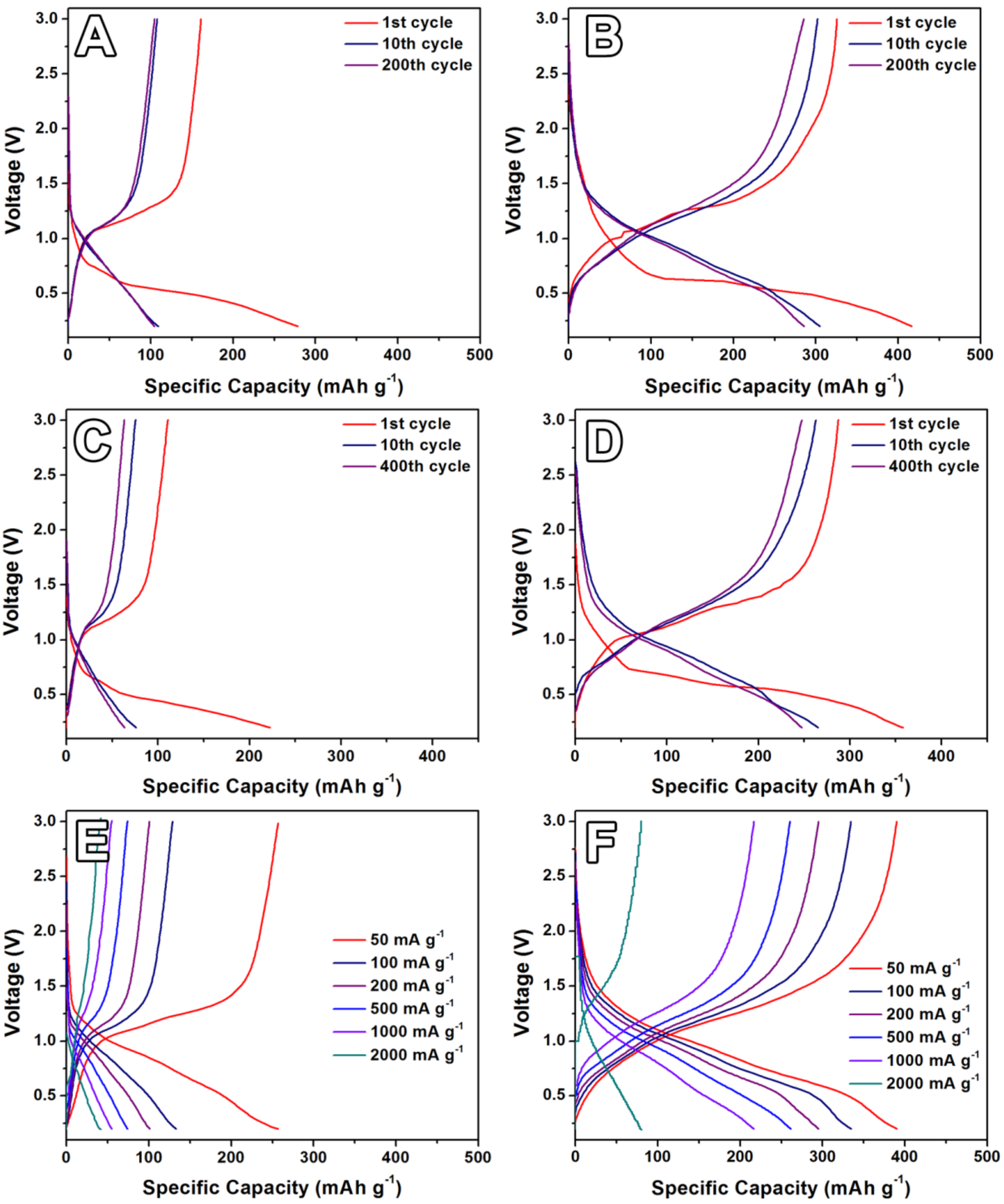

Figure S4. Galvanostatic charge-discharge profiles of Li_3VO_4 and $Li_3VO_{4-\delta}$ at a current density of 200 mA g^{-1} (A, B), 500 mA g^{-1} (C, D). The charge-discharge profiles of Li_3VO_4 and $Li_3VO_{4-\delta}$ at various current densities (E, F).

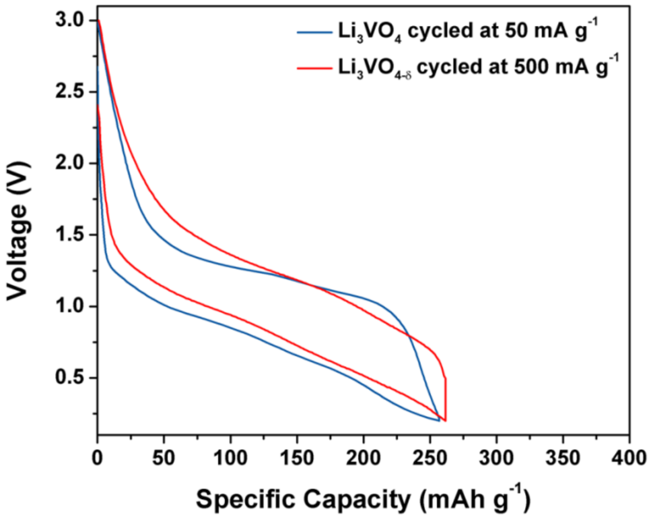

Figure S5. Comparison of galvanostatic charge-discharge profiles for the electrodes made of Li_3VO_4 and $Li_3VO_{4-\delta}$ cycled at 50 and 500 mA g^{-1}, respectively.

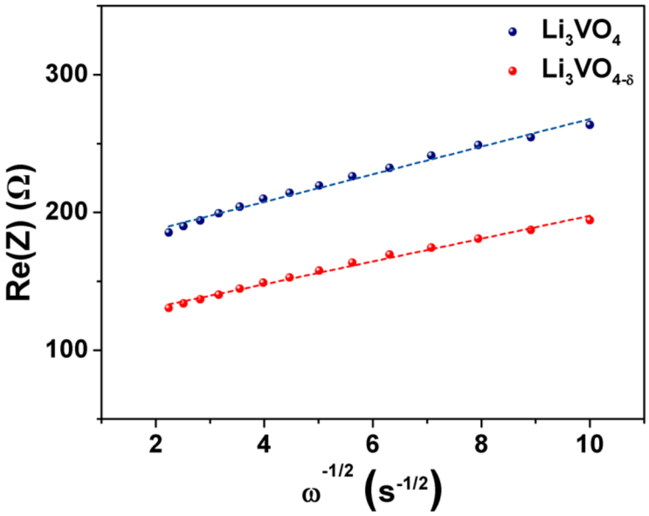

Figure S6. The plots of the real parts of the complex impedance versus $\omega^{-1/2}$.

The calculation for lithium diffusion coefficient in electrode materials is based on the following equation:

$$D = \frac{R^2T^2}{2A^2n^4F^4C^2\sigma^2}$$

where D is lithium diffusion coefficient, R is the gas constant (8.314 J mol^{-1} K^{-1}), T is the absolute temperature (298 K), A is the surface area of the electrode (~1 cm^2), n is the number of transferred electrons in the half-reaction, F is the Faraday constant (96485 C mol^{-1}), C is the concentration of Li ions in the solid (9.8×10^{-3} mol cm^{-3}) [1], and σ is the Warburg factor related to Z_{re} by this equation:

$$Z_{re} = R_D + R_L + \sigma\omega^{-1/2}$$

σ could be obtained by the linear fitting between Z_{re} and $\omega^{-1/2}$ (ω: the frequency), both of which could be obtained from EIS measurements. So, σ of Li_3VO_4 or $Li_3VO_{4-\delta}$ electrodes after five cycles is 11.2 or 9.0, corresponding to Li-ion diffusion coefficient at ~2.47×10^{-12} or ~3.82×10^{-12} cm^2 s^{-1}.
